# Training of formal caregivers in geriatric mental health and dementia care: Experiences and challenges from the Indian context

**DOI:** 10.1002/alz.087183

**Published:** 2025-01-09

**Authors:** Palanimuthu Thangaraju Sivakumar

**Affiliations:** ^1^ National Institute of Mental Health and Neurosciences, Bangalore India

## Abstract

The population of older adults in India is projected to increase from the current estimate of 150 million to 350 million by the year 2050. The prevalence of older adults with mental health problems including dementia is also increasing rapidly. The socio‐cultural changes in the joint family system have necessitated the increasing requirement of formal caregivers for supporting the care of older adults in home as well as residential care institutions. Home care services providing caregiver assistance, day care, old age homes, residential care institutions for persons with mental illness or dementia are available predominantly in major cities. However, there is a huge variability in the profile of formal caregivers ranging from those with no training to qualified nurses as caregivers depending on the affordability. The need for training of formal caregivers in geriatric mental health and dementia care is acknowledged by most of the the stakeholders in the care of older adults. However, there are no minimum standards of training for the formal caregivers. The lack of trained caregivers has contributed to the increase of elder abuse, higher stress, and burnout for the caregivers with adverse impact on the quality of care of older adults. This presentation will summarize the experience and challenges related to the implementation of the training of formal caregivers in geriatric mental health and dementia care. The training initiatives for the formal caregivers conducted by the Government and Non‐Governmental organizations ranges from brief sensitization program for half a day to formal certificate course for 3 months. Most of the training programs are focused on the basic aspects of geriatric care with inadequate component of practical training. High rates of discontinuation and change of jobs, poor working conditions, lack of motivation among the caregivers and employers, lack of mandatory requirement of training are some of the important challenges limiting the progress in ensuring trained formal caregivers.

